# Buruli ulcer: The Efficacy of Innate Immune Defense May Be a Key Determinant for the Outcome of Infection With *Mycobacterium ulcerans*

**DOI:** 10.3389/fmicb.2020.01018

**Published:** 2020-05-25

**Authors:** Katharina Röltgen, Gerd Pluschke

**Affiliations:** ^1^Department of Pathology, Stanford School of Medicine, Stanford University, Stanford, CA, United States; ^2^Medical Parasitology and Infection Biology, Swiss Tropical and Public Health Institute, Basel, Switzerland; ^3^University of Basel, Basel, Switzerland

**Keywords:** *Mycobacterium ulcerans*, skin neglected tropical disease, mycolactone, *Mycobacterium marinum*, zebrafish

## Abstract

Buruli ulcer (BU) is a neglected, tropical infectious disease of the skin and the subcutaneous tissue caused by *Mycobacterium ulcerans.* This pathogen has emerged as a new species from a common ancestor with *Mycobacterium marinum* by acquisition of the virulence plasmid pMUM. The plasmid encodes enzymes required for the synthesis of the macrolide toxin mycolactone, which has cytotoxic and immunosuppressive activities. In advanced BU lesions, extracellular clusters of *M. ulcerans* reside in necrotic subcutaneous tissue and are protected from infiltrating leukocytes by the cytotoxic activity of secreted mycolactone. Several lines of evidence indicate that elements of the innate immune system eliminate in many cases the initial inoculum before bacterial clusters can form and that therefore exposure to *M. ulcerans* leads only in a minority of individuals to the characteristic chronic necrotizing BU lesions. It is assumed that phagocytes play a key role in early host defense against *M. ulcerans*. Antibodies against bacterial surface structures seem to have less potential to enhance innate immunity than T_*H*_1 cell responses. Precise innate and adaptive immune effector mechanisms leading to protective immunity are however unclear, complicating the development of effective vaccines, the most desired solution to control BU. The tuberculosis vaccine *Mycobacterium bovis* Bacillus Calmette–Guérin (BCG) has limited short-term protective activity against BU. Whether this effect is due to the broad antigenic cross-reactivity between *M. bovis* and *M. ulcerans* or is at least partly mediated by a non-specific enhanced responsiveness of innate immune cells to secondary stimulation, recently described as “trained immunity” or “innate immune memory” is unknown but has major implications for vaccine design. Current vaccine research and development activities are focusing on recombinant BCG, subunit vaccines with selected *M. ulcerans* proteins, and the neutralization of mycolactone.

## Introduction

Innate immunity constitutes the first line of host defense against potentially pathogenic microbial invaders. It comprises physical and various chemical barriers, including antimicrobial proteins, to prevent entry into the host, as well as innate humoral (such as the alternative complement pathway) and cellular defense mechanisms that come into play if the epithelial barriers are breached. Innate immune cells, such as macrophages, neutrophils, and dendritic cells express pattern recognition receptors (PRRs), such as toll-like receptors (TLRs) that can detect so called pathogen-associated molecular patterns (PAMPs) common to many microorganisms, or damage-associated molecular patterns (DAMPs) of host molecules released by infected or dying cells. Upon recognition, these “sensor cells” can either act directly as effectors, phagocytosing and degrading the pathogens or indirectly, by producing inflammatory mediators, such as cytokines and chemokines that can attract and activate other immune cells. If the infection persists, phagocytes also connect the innate with the adaptive immune system by presenting antigens to antigen-specific T and B cells. Evidence has been accumulating in recent years that after infection or vaccination, innate immune cells display changes in their transcription programs and cell physiology, which may lead to transiently increased responsiveness upon secondary stimulation by microbial pathogens, a phenomenon termed “trained immunity” (Netea et al., 2011, 2016).

Pathogenic mycobacteria, including *Mycobacterium tuberculosis* and its near relative *M. marinum* (Stinear et al., 2008) have developed mechanisms to subvert the innate immune response. They can establish residence inside host macrophages and use host granulomas – organized immune cell aggregates, characterized by the presence of mature macrophages, that can contain but fail to eradicate infection foci – for their expansion and dissemination during the innate phases of infection (Ramakrishnan, 2013). *M. marinum*, which causes a granulomatous, tuberculosis-like disease in ectotherms (Tobin and Ramakrishnan, 2008), has gained popularity as a model organism for mycobacterial infections and has thus been extensively studied. Amongst different model systems, experimentally infected zebrafish (*Danio rerio*) embryos and early swimming larvae have become a powerful resource to study contributions of innate immune responses to combat mycobacterial infections (Davis et al., 2002). Due to the absence of an adaptive immune system at these early developmental stages, the zebrafish model has significantly advanced our understanding of innate host defense against mycobacterial infections.

In comparison, little is known on early interactions of the immune system with *M. ulcerans*, which causes the chronic, necrotizing skin disease Buruli ulcer (BU) and has emerged as a new species from a common ancestor with *M. marinum* by acquisition of a virulence plasmid (pMUM) and subsequent reductive evolution (Doig et al., 2012). Despite more than 98% overall nucleotide identity between the genomes of the two pathogens (Stinear et al., 2007), *M. ulcerans* has developed a markedly different strategy for immune evasion, primarily due to the pMUM-mediated ability to produce mycolactone, a diffusible cytotoxic and immunosuppressive macrolide toxin (George et al., 1999). While an early intra-macrophage growth phase of *M. ulcerans* has been postulated (Coutanceau et al., 2005; Silva et al., 2009), in advanced disease, *M. ulcerans* bacilli are predominantly found extracellularly in the necrotic core of BU lesions, that is devoid of living, infiltrating immune cells (Ruf et al., 2017). Infection with *M. ulcerans* can either be contained by the immune system as indicated by reports of spontaneous healing (Marion et al., 2016a; O’Brien et al., 2019) and of *M. ulcerans-*specific immune responses in exposed, but healthy individuals (Gooding et al., 2001; Diaz et al., 2006; Yeboah-Manu et al., 2012), or can lead to serious dermatologic manifestations and chronic necrotizing disease (Pluschke and Röltgen, 2019). Understanding of early immune mechanisms involved in the diverse outcome of infection with *M. ulcerans* is however incomplete. In this review article, we compare the pathogenesis of *M. ulcerans* and *M. marinum* infections and summarize current data on innate immune mechanisms against infection with *M. ulcerans*. Knowledge on correlates of protection against BU has important implications for the rational design of a vaccine – the ideal solution to control the disease as discussed at the end of this article.

## *Mycobacterium ulcerans* Has Evolved From an *M. marinum*-Like Progenitor

### Common Ancestry…

Buruli ulcer mainly affects inhabitants of rural, focal areas in West and Central Africa and yet the host range of *M. ulcerans* is broad (Röltgen and Pluschke, 2015). Apart from human BU lesions that most commonly involve the extremities, *M. ulcerans* has been isolated from lesions of other mammals in Australia (Fyfe et al., 2010) and from diseased fish and frogs around the world (Trott et al., 2004; Rhodes et al., 2005; Ranger et al., 2006; Stragier et al., 2008). Based on comparative genomic data, two major lineages of mycolactone-producing mycobacteria (MPM) have been distinguished, the classical lineage isolated from humans in Africa, Australia, and Papua New Guinea, and from other mammals and the ancestral lineage, which can be subdivided into at least two deep rooted sub-lineages; human disease isolates from Japanese patients (also designated *Mycobacterium ulcerans* subsp. *shinshuense*) and strains isolated from humans in the Americas and from ectotherms (Käser et al., 2007; Doig et al., 2012). Genomic data indicate that MPM have emerged only once through the acquisition of pMUM and therefore all MPM should be designated *M. ulcerans* (Yip et al., 2007; Pidot et al., 2010).

Proximity to aquatic habitats, and particularly activities within stagnant or slow flowing water bodies have been identified as a common risk factor for human BU in different geographical areas (Raghunathan et al., 2005; Kenu et al., 2014; N’krumah et al., 2017; Maman et al., 2018). Knowledge on transmission pathways and reservoirs of *M. ulcerans* is still fragmentary, but inoculation of the bacteria into the skin by postulated insect vectors or from environmental reservoirs via skin trauma is hypothesized. Association of *M. ulcerans* with aquatic environments has long been suspected due to its emergence from *M. marinum*, a ubiquitous pathogen of fish and other ectotherms. Occasional human *M. marinum* infections, which most commonly involve fingers and/or hands, are nowadays mainly connected with exposure to fish tanks, handling of fish, and boating/fishing-related activities (Johnson and Stout, 2015). Transmission of *M. marinum* is thought to occur through inoculation of the bacteria into the skin via cuts or lacerations (Petrini, 2006).

*Mycobacterium ulcerans* and *M. marinum* grow optimally at around 30°C and poorly at 37°C and above, which may at least in part explain their skin tropism and limited systemic dissemination. Both pathogens belong to the group of slow-growing mycobacteria, whereby the generation time in microbial culture medium of *M. ulcerans* (several days) is considerably longer than that of *M. marinum* (∼4–6 h) (Clark and Shepard, 1963; Marsollier et al., 2004). A mean incubation period of 4.5 months has been determined for *M. ulcerans* infections in a study on BU patients in Australia (Loftus et al., 2018). The incubation period of *M. marinum* is estimated to be ∼3 weeks, but can be up to several months long (Jernigan and Farr, 2000). Infections with both pathogens may resolve spontaneously by activities of the immune system but require long-term antibiotic treatment when established. Current WHO treatment recommendations for *M. ulcerans* infections comprise a combination therapy with rifampicin and clarithromycin (or streptomycin) for 8 weeks and surgery if indicated (World Health Organization, Global Buruli Ulcer Initiative, 2004; Phillips et al., 2020). There are no clinical trials to guide optimal management of *M. marinum* infections, but treatment with two active agents (clarithromycin/azithromycin, ethambutol, or rifampicin) for 3–4 months with adjunct surgical debridement for invasive infections has been reported (Griffith et al., 2007). Person to person transmission of *M. ulcerans* and *M. marinum* infection is considered unlikely. Thus, at first glance, characteristics of *M. ulcerans* and *M. marinum* and of the infections they cause seem very similar. Marked differences emerge however when comparing pathogenesis and host responses evoked by the two related mycobacterial species.

### … but Vastly Different Pathogenesis

Infection with *M. ulcerans* initially produces subcutaneous nodules or papules with a necrotic core or less frequently plaques and edema with laterally extended destruction of subcutaneous tissue. As the early stages of the disease are often painless, patients tend to report late to health facilities, when severe skin and soft tissue destruction has started. In advanced stages of the infection, the epidermis overlying the necrotic deeper layers of the skin sloughs off, and chronic ulcers with undermined edges develop. Histopathologic hallmarks of BU lesions are a progressive contiguous coagulative necrosis of the deep dermis and subcutaneous fat tissue with clusters of acid-fast bacilli (AFB), but no viable infiltrating leukocytes in the core of necrotic areas. In patients with chronic non-healing BU, squamous cell carcinoma may develop (Evans et al., 1999). Human *M. marinum* disease, often referred to as “fish tank granuloma” is commonly limited to a single, nodular cutaneous lesion, but can progress to invasive disease such as tenosynovitis and less frequently arthritis and osteomyelitis (Aubry et al., 2002; Johnson and Stout, 2015). The histopathological spectrum of *M. marinum* infections is broad and depends on the course and stage of the disease (Travis et al., 1985; Sia et al., 2016). Features in skin biopsies range from poorly formed granulomas with loose infiltrates of epithelioid macrophages, scattered multinucleated giant cells, and lymphohistocytic dermal inflammation to well-formed granulomas with circumscribed, nodular macrophage infiltrates. Granulomas frequently contain a central necrotic core, often surrounded by mixed inflammation and granulation tissue (Sia et al., 2016). In deep soft tissue and synovial biopsies, moderately well-formed, non-caseating or necrotizing, suppurative granulomas with giant cells are seen (Beckman et al., 1985; Sia et al., 2016). Acute and chronic synovial inflammation characterized by a paucity of plasma cells, often accompanied by synovial hyperplasia, and fibrin exudation into the synovial space has been reported (Beckman et al., 1985; Travis et al., 1985; Sia et al., 2016). The normal synovial architecture may be replaced by extensive granulation tissue (Travis et al., 1985). In both skin and synovial samples, bacilli are not easily detected as they reside highly localized in necrotic, suppurative cores of the granuloma (Sia et al., 2016). Variation in pathogenicity may also be related to genetic differences, as *M. marinum* strains exhibit extensive genomic diversity (van der Sar et al., 2004; Broutin et al., 2012). Despite an overwhelming sequence similarity of 98% between *M. ulcerans* and *M. marinum*, explanations for differences in pathogenesis and immune defense can be found in the gene content of their genomes.

### It Is Written in the Genes…

*Mycobacterium ulcerans* has acquired both a virulence plasmid, encoding genes for the biosynthesis of the unique macrolide toxin mycolactone, and insertion sequence (IS) elements that have mediated extensive loss of DNA. Whereas the genome of *M. marinum* strain M encompasses a 6.6 Mb circular chromosome with 5424 coding sequences (CDS), and 65 pseudogenes (Stinear et al., 2008), the genome of the *M. ulcerans* classical lineage reference strain Agy99 is considerably smaller, comprising the virulence plasmid pMUM of 174 kb, and a 5.6 Mb circular chromosome with 4160 CDS, and 771 pseudogenes (Stinear et al., 2007). Production of the macrolide toxin mycolactone was likely the key factor enabling the evolution of *M. ulcerans*.

**FIGURE 1:**
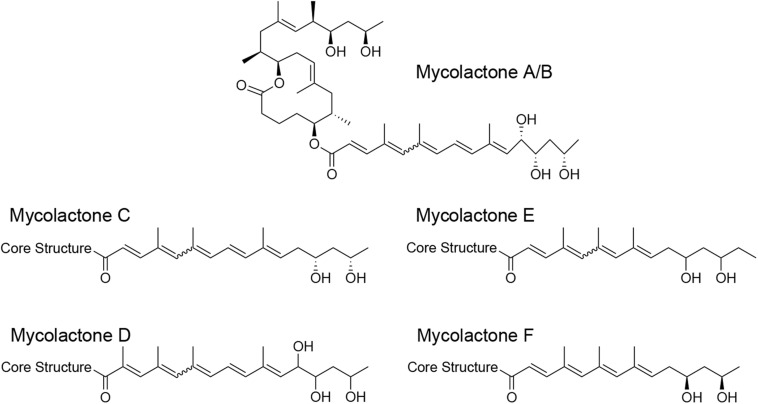
Structure of mycolactone variants. Mycolactone congeners were shown to be produced by *M. ulcerans* strains isolated from BU patients in Africa (mycolactone A/B), Australia (mycolactone C), and China (mycolactone D) and from fish and frogs of different geographical origin (mycolactone E and F).

Mycolactone consists of a conserved 12-membered lactone ring with a C-linked upper side chain and a less conserved lower C5-O-linked polyunsaturated acyl side chain (George et al., 1999). The lower side chains of the mycolactone variants produced by different *M. ulcerans* sub-lineages are structurally diverse (Figure 1) and differ in toxic potency (Scherr et al., 2013). In addition to potent cytotoxic activity, mycolactone exhibits analgesic and immunosuppressive properties at sub-toxic concentrations (Phillips et al., 2009; Hall et al., 2014; Marion et al., 2014; Guenin-Macé et al., 2019). Low nanomolar concentrations of mycolactone cause *in vitro* apoptosis within 2–5 days in a wide range of mammalian cells (Bozzo et al., 2010; Scherr et al., 2013; Guenin-Mace et al., 2015; Ogbechi et al., 2015). The secreted toxin seems to diffuse passively through mammalian cell membranes and to promote Bim-dependent apoptosis through the Akt-FoxO3 axis, as demonstrated by the absence of necrotic BU lesions in *M. ulcerans-*infected Bim knock-out mice (Bieri et al., 2017). In contrast, necrotizing lesions with features of human BU are caused by injection of mycolactone or mycolactone-producing *M. ulcerans* into the skin of wild-type mice and other experimental animals (George et al., 2000; Oliveira et al., 2005; Sarfo et al., 2013). Among several distinct proposed molecular mechanisms for the mode of action of mycolactone, selective inhibition of the Sec61 translocon-mediated co-translational transport of secretory proteins into the ER seems to play the key role (Hall et al., 2014; Baron et al., 2016). The inhibition of protein translocation leads to pronounced proteomic changes and an integrated cellular stress response that ultimately seems to drive Bim-dependent apoptosis. In addition, downregulation of cytokines and chemokines at sub-toxic concentrations has strong immunosuppressive effects. The Wiskott–Aldrich syndrome protein (WASP) family has been proposed as another molecular target of mycolactone (Guenin-Macé et al., 2013). Binding of the toxin to WASP/N-WASP appears to lead to uncontrolled assembly of actin and defects in cell adhesion, which may obstruct innate cellular immune responses.

One additional important difference in the gene content of the *M. marinum* and *M. ulcerans* genomes is the repertoire of ESX secretion systems and of PE/PPE proteins. The mycobacterial ESX loci are large gene clusters that encode a type VII secretory apparatus required for export of members of the 6-kDa early secreted antigenic target (ESAT-6) protein family together with other effector proteins across the complex cell envelope. Genes encoding ESAT-6 and the 10 kDa culture filtrate protein (CFP-10) are located directly adjacent to each other and are co-transcribed. The genome of the *M. tuberculosis* H37Rv strain contains 11 pairs of these *esx* tandem genes (Cole et al., 1998). *M. marinum* has 29 *esx* genes within five ESX loci (Stinear et al., 2008). The prototypical ESX-1 system is a major virulence determinant in *M. tuberculosis* and *M. marinum*, triggering granuloma formation as well as intercellular bacterial spread between macrophages (Volkman et al., 2004). The absence of a 9.5 kb genomic region across all *Mycobacterium bovis* Bacillus Calmette–Guérin (BCG) strains, termed Region of Difference 1 (RD1), located in the ESX-1 locus, is a major molecular determinant underlying BCG attenuation. *M. marinum* carries a partial duplication of the *esx-1* gene cluster, resulting in more than one copy of several genes including *esxA* (ESAT-6) and *esxB* (CFP-10). On the other hand, *M. ulcerans* has retained only 13 *esx* genes and three intact ESX loci. Disruption of the ESX-1 locus in *M. ulcerans* classical lineage strains, which led to the abolishment of ESAT-6 and CFP-10 secretion (Huber et al., 2008), may contribute to the predominantly extracellular location of *M. ulcerans* bacilli (Stinear et al., 2007).

PE/PPE proteins, found mostly in slow-growing pathogenic mycobacteria, are characterized by conserved Pro-Glu (PE) and Pro-Pro-Glu (PPE) motifs at the N-termini. It has been reported that certain ESX secretion systems mediate the secretion of several PE/PPE proteins (Abdallah et al., 2009; Shah et al., 2015). The genome of the *M. tuberculosis* H37Rv strain contains 99 *pe* and 69 *ppe* genes, but this number can vary for different *M. tuberculosis* isolates (Fishbein et al., 2015). The *M. marinum* genome codes for 175 PE and 106 PPE proteins, whereas *M. ulcerans* has preserved only 70 intact *pe* and 46 *ppe* genes. The function of PE and PPE proteins is still enigmatic, but the limited data available suggest that they are important for mycobacterial virulence. Some of the PE proteins from *M. marinum* are thought to be involved in modulating the macrophage environment (Ramakrishnan et al., 2000; Tiwari et al., 2012; Fishbein et al., 2015). Compared to *M. marinum, M. ulcerans* has thus both gained a major virulence determinant – mycolactone and lost several other virulence factors mainly associated with the intracellular lifestyle of its ancestor. The following two paragraphs illustrate how these genomic differences are reflected in diverse interactions of *M. marinum* and *M. ulcerans* with the innate immune system.

## Host-Mycobacterium Interactions – Insights Into Early Infection Events From a Zebrafish Model

In the past decades, *M. marinum* has become a model organism to study fundamental mechanisms of mycobacterial pathogen-host interactions. Zebrafish are naturally susceptible to *M. marinum* and upon infection develop organized granulomas similar to those caused by *M. tuberculosis* (Westerfield, 2000; Swaim et al., 2006). Zebrafish embryos and early swimming larvae are a powerful means to dissect innate immune responses to *M. marinum*, as at these early developmental stages, they rely solely on innate immune mechanisms mediated by macrophages and neutrophils, and lack the elements of adaptive immunity (Davis et al., 2002). In this paragraph, we describe how the genetic tractability and optical transparency of zebrafish embryos have enabled a variety of elegant experimental approaches to study early *M. marinum* infection events *in vivo*.

### First Hours After Infection

Real-time imaging of zebrafish embryos revealed that after intravenous injection of *M. marinum*, blood macrophages immediately take up mycobacteria and extravasate into diverse tissues. Strikingly, recruitment of macrophages within 6 h after injection of *M. marinum* into the zebrafish hindbrain ventricle – an isolated cavity devoid of macrophages in the absence of pathogens – demonstrated redirection of normal migration and differentiation of embryonic macrophages (Davis et al., 2002). Both live and heat-killed *M. marinum* seem to be able to recruit phagocytes (heat-killed mycobacteria are subsequently readily degraded after phagocytosis), suggesting that cell wall lipids or heat-stable proteins stimulate migration (Clay et al., 2007). It has been postulated that *M. marinum* is able to evade microbicidal effects of TLR-activated macrophages by masking PAMPs with cell-surface associated lipids, and instead recruits permissive macrophages via an alternative pathway. In infected macrophages, the production of inflammatory cytokines, such as tumor necrosis factor (TNF)-α and interleukin (IL)-1β is upregulated (Cambier et al., 2014). Studies involving the depletion of macrophages in zebrafish embryos illustrated that macrophages are required to restrict proliferation of *M. marinum* and thus constitute only a suboptimal growth niche for the bacilli. They do however play an important role in the dissemination of mycobacteria into the tissues (Clay et al., 2007).

### Migration Into Tissue and Granuloma Formation

After phagocytosing the *M. marinum* bacilli, macrophages migrate into deeper tissue, where they begin to form dynamic, granuloma-like aggregates, becoming visible three days after intravenous inoculation. Aggregated cells display euchromatic nuclei characteristic of activated macrophages and either tightly apposed cell membranes or indistinct cell boundaries, distinctive features of epithelioid cells and multinucleated giant cells in mature granulomas. *M. marinum* is found both intracellular, sequestered by the cellular aggregates, or extracellular, in necrotic (caseous) foci (Davis et al., 2002). TNF was found to be a key effector molecule required for the maintenance of granuloma integrity. Ablation of TNF signaling in mutant zebrafish embryos causes both accelerated intracellular bacterial growth and granuloma formation, followed by increased macrophage death and necrotic breakdown of granulomas with resultant exuberant growth of extracellular mycobacteria (Clay et al., 2008). Interestingly, granuloma formation is generally associated with accelerated bacterial proliferation, plateauing only after several weeks with the onset of adaptive immunity, indicating that nascent granulomas promote mycobacterial expansion (Volkman et al., 2004). Indeed, zebrafish studies have illustrated that granuloma-forming processes are mediated through mycobacterial virulence factors. By a mechanism requiring the mycobacterial RD1/ESX-1 secretion system, new macrophages are recruited to the granuloma via chemotactic signals, and phagocytose infected macrophages undergoing apoptosis, leading to rapid, iterative expansion of the number of infected macrophages and thereby to an increase in the bacterial burden (Davis and Ramakrishnan, 2009). The secretory protein ESAT-6 seems to play a central role in these chemotactic effects by inducing matrix metalloproteinase-9 (MMP-9) expression in epithelial cells surrounding the granuloma. In turn, MMP-9 is thought to enhance recruitment of macrophages to the growing granuloma. RD1/ESX-1-deficient *M. marinum* strains are still able to recruit macrophages to the infection site, but fail to elicit aggregation into granulomas and intercellular bacterial spread and thus presumably lack the ability to induce chemotactic signals required for the initiation of these processes (Volkman et al., 2004). Primary granulomas can seed new granulomas by the efflux of infected macrophages, constituting a means of disseminating infection (Davis and Ramakrishnan, 2009).

### The Role of Neutrophils

Macrophages have been described as the predominant cell type phagocytosing *M. marinum* after microinjection into fluid-filled compartments, such as the blood or hindbrain ventricle. In contrast, *M. marinum* seems to evade contact with neutrophils at initial infection sites. Instead, neutrophils are subsequently recruited to the nascent granuloma in response to signals from dying infected macrophages within the granuloma. Neutrophils are able to phagocytose and rapidly kill the internalized mycobacteria through NADPH oxidase-dependent mechanisms (Yang et al., 2012). Interestingly, effective uptake of *M. marinum* by neutrophils has been observed after subcutaneous infection of zebrafish (Colucci-Guyon et al., 2011). Likewise, it was found in the zebrafish model of *Escherichia coli* infection that neutrophils efficiently engulf bacteria on tissue surfaces, but not in fluid environments (Le Guyader et al., 2008). The concept that the route of entry of the bacteria into the host may determine the role of neutrophils in infections may also be of relevance for human infectious diseases, particularly for the early phase of encounter with microbes (Colucci-Guyon et al., 2011).

### From Initial Granuloma Formation to Mature Granulomas

**FIGURE 2:**
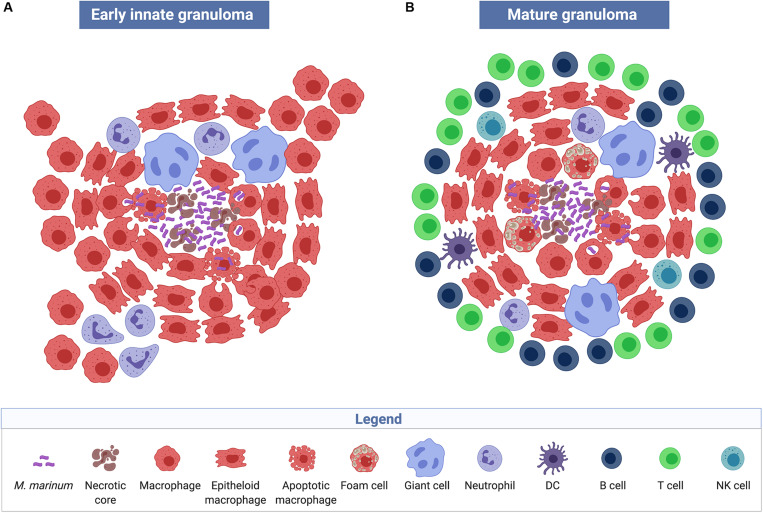
Structure and cellular composition of granulomas. Early innate *M. marinum* granulomas, as described in zebrafish embryos, are composed of compact, organized aggregates of macrophages that have either transformed into epithelioid cells with tightly interdigitated cell membranes or have fused to become multinucleated giant cells. Bacilli are found both extracellularly in the necrotic core of the granuloma and intracellularly within phagocytes **(A)**. The characteristic mature tuberculous granuloma is populated by many other cell types such as neutrophils, dendritic cells, B and T cells, natural killer cells, and foam cells, which are macrophages with accumulated lipids **(B)** (created with biorender.com).

The view that granulomas are primarily host-beneficial protective structures, is thus challenged by the described findings in the zebrafish embryo model of *M. marinum* infection. Results demonstrate that pathogenic mycobacteria have developed mechanisms to harness nascent host granulomas for their dissemination and proliferation (Ramakrishnan, 2013). Pathologically, granulomas are defined as organized collection of differentiated macrophages with characteristic morphology, such as epithelioid histiocytes and giant cells (Figure 2A). In addition to macrophages, mature tuberculous granulomas in humans are populated by many other cell types, including neutrophils, dendritic cells, B and T cells, natural killer cells, fibroblasts, and epithelial cells (Figure 2B). The role of these cells in granulomatous infectious diseases is yet to be fully elucidated. Another characteristic of certain tuberculous granulomas is the presence of regions of acellular debris referred to as caseous necrosis. This limited central necrosis is also found in *M. marinum* infection; mycobacteria can be located both within macrophages and within the central caseous region. Microbial virulence factors may influence the cellular composition of granulomas and the role of these structures played in either the containment, persistence, or dissemination of infections.

## Innate Immune Mechanisms in *M. ulcerans* Infection

### Entry Point: Skin

The site of *M. ulcerans* and *M. marinum* lesions is thought to also be the site of inoculation of the mycobacteria into the skin, as indicated by the vast majority of patients presenting with a single skin lesion on body parts that are likely exposed to the contaminated environment and/or, in the case of *M. ulcerans*, to potential vectors (Portaels et al., 1999; Johnson et al., 2007; Lavender et al., 2011). Definitive evidence for this assumption is however lacking. For both mycobacterial skin infections, the skin epithelium constitutes the first physical barrier. In healthy skin, stable homeostasis and barrier function are established by resident keratinocytes, dendritic cells, T cells, mast cells, fibroblasts, and macrophages together with the resident microbiome (Gallo and Nakatsuji, 2011). Contact with environmental reservoirs of *M. ulcerans* or *M. marinum*, and penetration of the bacilli into the subcutaneous tissue through trauma – in the case of *M. ulcerans* potentially including bites by contaminated insects acting as mechanical vectors (Wallace et al., 2017) – may be the most common mechanism of infection, although other mechanisms cannot be excluded. The outcome of an infection with both *M. ulcerans* and *M. marinum* may depend on the mode of transmission and the initial dose of inoculated bacteria. In a guinea pig infection model, it has been shown that BU can be produced by intra-dermal injection, but not through inoculation of *M. ulcerans* onto a superficial abrasion (Williamson et al., 2014). In advanced BU lesions, clusters of extracellular AFB are predominantly found in deep layers of the subcutaneous fat tissue (Ruf et al., 2016). This may reflect the location of the initial inoculum causing disease or the presence of a microenvironment in the necrotic tissue which is most favorable for the multiplication of *M. ulcerans.* In a case series of patients with *M. marinum* infection, boating or fishing were associated with invasive disease, whereas fish tank exposure was associated with cutaneous disease. This may be related to the mechanism of injury; boating and fishing injuries may involve deep puncture wounds from fish spines, fishhooks or other equipment, while fish tank injuries may involve more superficial exposures such as minor scrapes while cleaning or maintaining fish tanks (Johnson and Stout, 2015).

### Once Inside the Host…

Once inside the host, the mycobacteria encounter innate immune cells expressing various PRRs. Based on a series of *in vitro* experiments it was postulated that keratinocyte TLRs may play a role in the innate immune response to *M. ulcerans* infections (Lee et al., 2009). The precise role (if any) of keratinocytes in the recognition of *M. ulcerans* and subsequent modulation of innate responses in the host is however unknown. If we assume that the mode of transmission of the mycobacteria allows for an encounter with these cells, keratinocyte TLRs, in response to sensing PAMPs expressed by microbes and DAMPs produced by the host, can initiate an inflammatory cascade including the release of inflammatory cytokines and host antimicrobial molecules. Early events in the innate immune response to skin injury include the recruitment of neutrophils via chemokines released by the keratinocytes. Later during the inflammatory cascade, macrophages are the predominant immune cell type (Coates et al., 2018). At this stage of the infection, the ability to produce mycolactone results in a fundamentally different interaction of the host with *M. ulcerans* as compared to *M. marinum*.

### Insight Into Early Immune Defense Against *M. ulcerans* From Animal Models

To study immune responses setting in directly after infection with *M. ulcerans*, experimental infection models mostly involving mice (*Mus musculus*), but also other animals such as guinea pigs (*Cavia porcellus*) or pigs (*Sus scrofa*) have been developed (Bolz and Ruf, 2019). In the mouse model of BU, an immediate massive influx of neutrophils and to a lesser extent of monocytes/macrophages at the site of *M. ulcerans* intraperitoneal or intradermal injection is observed. One day after injection, bacilli are found within phagocytes and some of the inoculated bacilli are subsequently transported to the draining lymph nodes, where T_*H*_1-type cellular immune responses are initiated. Prompted by these findings, an early intracellular growth phase of *M. ulcerans* that induces inflammatory cellular responses has been postulated (Coutanceau et al., 2005; Oliveira et al., 2005; Torrado et al., 2007; Silva et al., 2009). As early as 24 h after injection of *M. ulcerans* into mouse footpads, lysis of infected phagocytes mediated by the production of mycolactone by *M. ulcerans* causes release of the bacteria into the extracellular space (Oliveira et al., 2005). In particular globi-like accumulations of bacilli released from phagocytes (Schütte et al., 2009; Ruf et al., 2011) may readily form a protective cloud of mycolactone and may represent starting points for the development of large extracellular clusters. During this second stage of the infection, bacteria can multiply predominantly extracellularly, as mycolactone appears to prevent infiltrating immune cells from reaching the mycobacteria (Ruf et al., 2017). *M. ulcerans* forms an extracellular matrix, which is rich in proteins, lipids and lipoglycans and is likely to play a role in the development of extracellular clusters (Marsollier et al., 2007). High concentrations of mycolactone in the lesion core cause apoptosis of both resident skin cells and infiltrating leukocytes. Chronic, necrotic lesions develop upon the invasion of the bacteria into healthy tissue and the progressive lateral destruction of subcutaneous tissue (Oliveira et al., 2005). Ischemia associated with vascular pathology may also contribute to the coagulative necrosis. In the infiltrate surrounding the necrotic core, intracellular bacilli can be detected, mainly in macrophages (Oliveira et al., 2005; Torrado et al., 2007). In the experimental mouse footpad infection model, dermal edema and footpad swelling become – dependent on the inoculation dose – evident a few weeks after infection. Necrosis extends to components of subcutaneous tissue, eventually resulting in extensive ulceration of the epidermis. That the continuous expansion of necrotic lesions is mainly mycolactone-mediated is illustrated in infections with mycolactone-negative *M. ulcerans* strains, which induce an initial acute neutrophilic response that gradually switches to a chronic mononuclear infiltrate devoid of massive necrosis (Oliveira et al., 2005).

**FIGURE 3:**
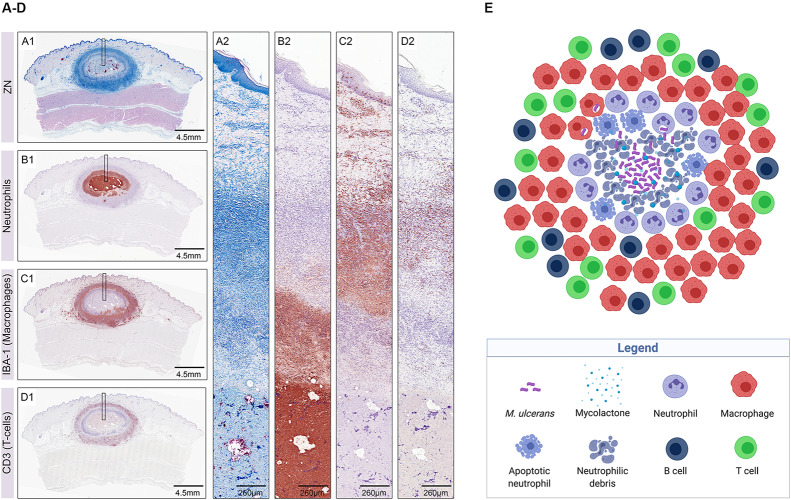
Early host immune response to *M. ulcerans* infection illustrated in histological sections of a nodular lesion 6 weeks after subcutaneous infection of pigs **(A–D)** (from Bolz et al., 2016b) and in a sketch depicting the cellular composition of the lesion **(E)** (created with biorender.com). Histopathological analysis of pig lesions shows a central necrotic core containing extracellular *M. ulcerans* bacilli (**A1,A2**; AFB stained with Ziehl–Neelsen staining) and neutrophilic debris **(B1,B2)**. The necrotic core is surrounded by layers of neutrophils **(B1,B2)** and macrophages **(C1,C2)**, heavily interspersed with T-cells **(D1,D2)**.

The pig is widely used as a model in dermatological studies because pig and human skin share many morphological and physiological features. Pigs have been shown to develop single lesions at the site of injection of *M. ulcerans* into the skin, characterized by a central necrotic core containing large clumps of AFB surrounded by layers of neutrophilic debris, some intact neutrophils, and an outer belt of macrophages interspersed with T cells (Figures 3A–E). In contrast, lesions caused by mycolactone-deficient *M. ulcerans* strains present as multiple small central clusters of neutrophils and AFB with only limited necrosis, surrounded by a massive infiltration of macrophages interspersed with T-cells (Bolz et al., 2016b).

Interestingly, both wild type and mutant *M. ulcerans* strains evoke the same sequential infiltration layers with neutrophils and neutrophilic debris in the necrotic lesion centers surrounded by macrophages and T cells (Bolz et al., 2016b), a composition which is in contrast to granulomatous lesions caused by *M. marinum* or *M. tuberculosis*, characterized by organized aggregates of mature macrophages.

### Insight Into Early Immune Responses From the Study of Early Human BU Lesions

Buruli ulcer patients in rural, endemic areas of Africa typically report to health facilities at late stages of the disease and thus histopathological studies are mostly restricted to advanced ulcers. In a cohort of 12 BU patients from far north Queensland (Australia) presenting with early lesions, immunohistochemical analysis revealed an acellular, necrotic core containing the extracellularly multiplying AFB separated from intact tissue by a belt of infiltrating immune cells comprising clusters of CD20-positive B cells, CD3-positive T cells, neutrophils, and macrophages. Neutrophilic debris was found inside the lesion core and is indicative of a massive early neutrophil infiltration that was walled off by the cytotoxic actions of mycolactone (Figure 3E). Some AFB, located close to the infiltration belt, were detected intracellularly and may be signs of an expansion of the necrotic foci into healthy tissue (Ruf et al., 2017), a finding that was also reported in another study (Torrado et al., 2007). Although the pathogenesis of *M. ulcerans* infections in Australia and Africa seems comparable, it remains to be investigated if similar early immune responses are evoked in BU patients from Africa.

### Immune Reconstitution After Antibiotic Therapy

**FIGURE 4:**
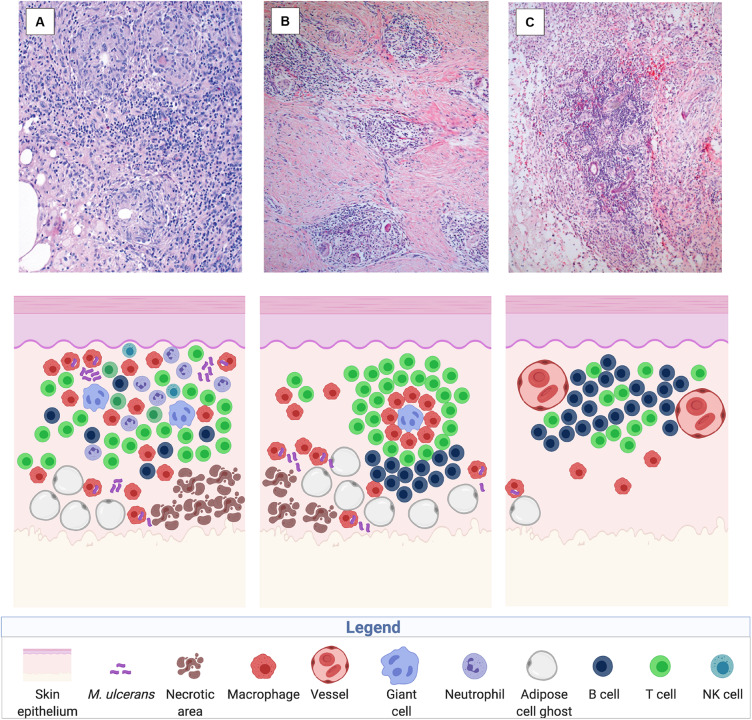
Immune reconstitution responses after chemotherapy illustrated in histological sections of a human BU lesion (from Schütte et al., 2007) and in a sketch of the participating immune components (created with biorender.com). Three main types of cellular infiltration have been observed in BU patients after treatment with the standard antibiotic regimen, including diffuse, heterogeneous cellular infiltration of the connective and adipose tissue (100× magnified) **(A)**, granuloma-like structures in the connective tissue (40× magnified) **(B)**, and follicle-like lymphocyte structures adjacent to vessels (40× magnified) **(C)**.

Antibiotic treatment of BU patients results in a rapid onset of local cellular immune responses. Analysis of surgical specimens excised from patients after treatment with the standard antibiotic regimen for BU, revealed accumulation of infiltrating leukocytes in the BU lesions, presumably facilitated by decreasing concentrations of mycolactone associated with the suppression of the metabolic activity and finally with the killing of the bacilli. Already 4 weeks after start of antibiotic treatment AFB are primarily located within mononuclear phagocytes. Cellular infiltrates surrounding areas of coagulative necrosis display different levels of organization, including diffuse infiltrates present in all areas of connective and adipose tissue (Figure 4A) and, less frequently, organized epithelioid leukocyte accumulations located in deeper dermal tissue (Figure 4B) or dense lymphocyte aggregations in proximity to vessels (Figure 4C), reflecting a range of different functional activities required to clear the infection (Schütte et al., 2007).

### Diverse Outcome of Infection

The early immune response may in many cases be capable of clearing an initial *M. ulcerans* inoculum. Spontaneous healing of BU lesions and serological evidence of exposure of healthy individuals to *M. ulcerans* indicate that the host immune system can contain infections with the pathogen, although mechanisms conferring protection are not entirely clear. Progression of lesions is highly diverse and not all non-ulcerative lesions ulcerate (Capela et al., 2015). One factor for the diverse outcome of *M. ulcerans* infections may be the inoculation dose. In the pig model of *M. ulcerans* infection, lower inoculation doses led to limited tissue destruction and eventually to the clearance of the bacteria (Bolz et al., 2014). Healing of BU lesions without specific treatment has been described for nodules, small ulcers, and long-standing ulcerative lesions (Revill et al., 1973; Marion et al., 2016a; O’Brien et al., 2019). How frequently BU lesions heal spontaneously is however difficult to assess, as many patients with early stages of the disease, that are usually indolent and non-systemic, may not report to health facilities. In a series of 545 BU patients diagnosed at a BU treatment center in Benin, 5% of the cases healed without specific treatment (Marion et al., 2016a). Whereas subcutaneous injection of *M. ulcerans* into BALB/c or C57BL/6 mice eventually leads to irreversible ulceration and tissue necrosis, ulcerative lesions developing after infection of FVB/N mice with *M. ulcerans* healed spontaneously despite persistent bacterial load. The healing process in FVB/N mice was associated with an infiltration of predominantly mononuclear cells, such as macrophages, dendritic cells, and neutrophils at the site of infection and an accumulation of myeloid cells in the draining lymph nodes, suggesting an important role for innate cellular immune defense mechanisms in protection (Marion et al., 2016b). In contrast to FVB/N mice, experimentally infected guinea pigs and pigs are able to entirely clear the *M. ulcerans* bacilli during the process of spontaneous healing (Bolz et al., 2014, 2016b; Silva-Gomes et al., 2015).

Children living in BU endemic areas of Africa seem to gradually develop resistance against BU with age, as indicated by a decline in the risk to develop the disease after a peak at an age of 12–14 years (Debacker et al., 2004, 2006; Bratschi et al., 2013). An increase in risk to develop BU in the elderly may be related to a deterioration of the relevant immune defense mechanisms. Prevalence of human immunodeficiency virus (HIV) infection in BU patients is significantly higher than in the local control population (Johnson et al., 2008; Christinet et al., 2014), indicating that T_*H*_1 cell responses are important enhancers of the innate cellular immune defense against *M. ulcerans* infection. Moreover, skin lesions in BU-HIV co-infected patients tend to be more severe and more often multifocal (Johnson et al., 2002; Komenan et al., 2013). Case control studies aiming at the identification of host genetic factors relevant for susceptibility to BU have focused so far primarily on polymorphisms in genes known to be relevant for intracellular mycobacterial infections (Stienstra et al., 2006; Capela et al., 2016; Bibert et al., 2017). Significant associations observed with susceptibility to BU include polymorphisms affecting the promoter activity of the *IFN-γ* gene, the inducible nitric oxide synthase gene *iNOS* and the natural resistance-associated macrophage gene *SLC11A1* (*NRAMP1*). These results for the primarily extracellular *M. ulcerans* support the view that macrophages are crucially important for the early containment of *M. ulcerans* infections. Diversity of the response to human infection may thus be influenced not only by factors like inoculation dose, age, and nutritional status, but also by a complex constellation of genetic factors.

## Conclusion

Despite common ancestry and the high degree of genetic relatedness of *M. marinum* and *M. ulcerans*, the role of the innate host immune system in immune defense against the two pathogens seems to differ considerably. Macrophages are a key component in the innate immune response to *M. marinum* infection, where they inadvertently play a dual role, both containing mycobacterial growth and providing an environment where the bacilli can persist (Ramakrishnan, 2012). Continuous recruitment of macrophages (and other immune cells), at least partly dependent on mechanisms of the RD1/ESX-1 secretion system, leads to a characteristic localized inflammatory response and granuloma formation. On the contrary, phagocytes seem to be only transiently inhabited by *M. ulcerans* in very early stages of the infection (Coutanceau et al., 2005). Histopathological analyses of human BU lesions and of those of experimentally infected animals indicate that neutrophils are the main infiltrating cell type in early stages of *M. ulcerans* infection, later complemented by macrophages (Bolz et al., 2016b; Ruf et al., 2017). The early dominance of neutrophils may be explained by the route of *M. ulcerans* infection (Le Guyader et al., 2008; Colucci-Guyon et al., 2011). After a postulated short intra-phagocyte stage of *M. ulcerans*, production of mycolactone enables a second, extracellular growth phase. Bacterial clusters are formed, which produce a protective cloud of mycolactone, walling off and killing infiltrating immune cells. A progressive invasion of healthy tissue and potentially of phagocytes at the periphery of the lesions leads to the characteristic chronic, necrotic course of the disease (Ruf et al., 2017). In BU patients under chemotherapy, killing of the bacteria and consequently the decrease in mycolactone concentrations allows for an immune reconstitution reaction (Schütte et al., 2007).

Due to relatively unspecific first signs of *M. ulcerans* infections, the often indolent course of the disease, and the limited access of affected populations to medical care, patients commonly seek treatment primarily in advanced disease stages, leaving patients in many cases with permanent disabilities. This and the highly focal occurrence of the disease makes vaccination of inhabitants of endemic areas the desired solution for BU control, particularly because the limited understanding of risk factors for BU and reservoirs/vectors of *M. ulcerans* have so far hindered other preventive measures. Lack of knowledge on protective host immune responses has however also complicated the design of a vaccine for BU. Intriguingly, there is striking paucity of humoral immune responses upon infection with *M. ulcerans*, revealed after experimental *M. ulcerans* infection in the BU mouse model (Bieri et al., 2016; Bolz et al., 2016a), and corroborated by reports that sera of only a minority of BU patients contain *M. ulcerans*-specific antibodies (Yeboah-Manu et al., 2012). These findings may be explained by the cytotoxic and immunosuppressive actions of mycolactone on immune cells. This and evidence pointing toward an early intracellular phase of *M. ulcerans* call for a robust engagement of innate immune cells to boost cell-mediated immunity, eliminating the phagocytosed mycobacteria before larger intra- and later on extra-cellular toxin-producing bacterial clusters can form. Efficient activation of T cells orchestrated by antigen presenting cells is crucial to enable in turn activation (CD4 T cells) of host cells, such as macrophages to kill invading pathogens or direct killing (CD8 T cells) of infected host cells. The specific types of mycobacterial T cell antigens conferring protective immunity are yet to be determined. In addition to adaptive T cells activated upon mycobacterial peptide antigens presented by major histocompatibility complex (MHC) molecules, innate-like T cells with low antigen receptor diversity, which recognize lipid antigens presented by cluster of differentiation 1 (CD1), have been described (Beckman et al., 1994). T cells recognizing mycobacterial glycolipid antigens, which share some biological properties with both adaptive and innate-like T cells were shown to confer protection to tuberculosis in animal models (Larrouy-Maumus et al., 2017; James and Seshadri, 2020). In the past, strategies for the development of vaccines have mainly been empirical, with limited understanding of the underlying immune mechanisms, using killed or live attenuated forms of the pathogens. Live-attenuated vaccines such as BCG in the case of *M. tuberculosis* seem to be suitable for intracellular pathogens as they induce a broad range of immune responses including strong CD8 T cell responses. Interestingly, it has recently been reported that a prolonged increase in antimicrobial function of innate immune cells can itself contribute to protection from reinfection. In this context it was shown that BCG is capable of inducing non-specific cross-protection against microbes, a phenomenon that has been associated with a memory-like response in innate immune cells (Netea et al., 2011, 2016; Koeken et al., 2019). Such “trained innate immune cells” display functional and epigenetic reprogramming, leading to increased production of cytokines and chemokines, and improved phagocytotic and killing activities. Indeed, BCG was shown to offer a short-lived protective effect against BU in the first year after vaccination but limited to no protection thereafter (The Uganda Buruli Group, 1969; Smith et al., 1976). Whether this observed effect is due to mechanisms of trained immunity or is based on the broad antigenic cross-reactivity between *M. bovis* and *M. ulcerans* is however unknown. Similar results were found in the mouse model of experimental *M. ulcerans* infection, where BCG was shown to induce an immune response transiently containing proliferation of the bacilli but ultimately failing to prevent the typical BU pathology (Fraga et al., 2012). Differences in the effectiveness of BCG vaccination in different mouse strains has been reported (Converse et al., 2011). Genetically engineered BCG developed as a vehicle for BU vaccines offered marginally improved protection in the mouse model of *M. ulcerans* infection (Hart et al., 2015; Hart and Lee, 2016).

Considering that successful toxoid vaccines such as those against diphtheria and tetanus exist, targeting mycolactone itself may be a promising approach for the development of a BU vaccine. The presence of pre-existing neutralizing antibodies against mycolactone may physically block toxin interactions with host cells and thus aid cellular immune responses to the pathogen. Immunization of mice with a carrier protein conjugate of a non-toxic, synthetic mycolactone derivative has enabled for the first time the generation of antibody responses against the poorly immunogenic, cytotoxic mycolactone. Intriguingly, mycolactone-specific immune sera and mycolactone-specific mouse monoclonal antibodies showed toxin neutralizing activity, preventing mammalian cell apoptosis in an *in vitro* assay (Dangy et al., 2016). A more holistic approach targeting protective immune responses against both mycolactone and against other protein and potentially also glycolipid antigens may improve protective efficacy. To increase immunogenicity by activating appropriate elements of the innate immune system such a vaccine may need to be formulated with adjuvants stimulating various PRRs on innate immune cells (Tima et al., 2016).

Better understanding of the exact innate and adaptive immune mechanisms leading to protection from BU will help in the development of new strategies for effective vaccine design.

## Author Contributions

GP was invited to writing the review. KR and GP drafted and revised the manuscript.

## Conflict of Interest

The authors declare that the research was conducted in the absence of any commercial or financial relationships that could be construed as a potential conflict of interest.
